# Cadaveric Measurements of the Left Recurrent Laryngeal Nerve, Ligamentum Arteriosum, Aortic Arch, and Pulmonary Artery in the Thorax with Clinical Implications and Comparison Between Two Sexes in the American Population

**DOI:** 10.7759/cureus.4828

**Published:** 2019-06-04

**Authors:** Anasuya Ghosh, Subhramoy Chaudhury

**Affiliations:** 1 Anatomy, Medical University of the Americas, Charlestown, KNA

**Keywords:** left recurrent laryngeal nerve, aortic arch diameter, pulmonary artery diameter, ligamentum arteriosum, vocal cord palsy

## Abstract

The left recurrent laryngeal nerve (RLN) is prone to get compressed or damaged, leading to vocal cord palsy, due to pathologies or surgeries of the structures closely surrounding this nerve in the thorax, including the esophagus, aortic arch, pulmonary trunk, and ligamentum arteriosum. We wanted to provide a data set including nerve diameter, its distance from the esophagus, measurements of the pulmonary artery, aorta, and ligamentum arteriosum in close proximity of the nerve in a healthy population to avoid its damage during surgery and predict its chances of compression during the diseased condition. We measured the left RLN and the surrounding structures in 39 well-embalmed cadavers. We compared the values among the male and female cadavers. We found that the mean diameter of the left RLN was 1.75 mm, the mean distance of the nerve from esophagus was 9.88 mm, the mean diameters of the aortic arch and pulmonary artery just distal to the attachment of the ligamentum arteriosum were 26.14 and 19.93 mm, respectively, and the length and width of ligamentum arteriosum were 15.89 and 2.79 mm, respectively. No clinically significant differences were found between male and female parameters. This set of values might be useful while investigating the cause of vocal cord palsies or during surgeries in close proximity to left RLN to avoid its damage.

## Introduction

The left recurrent laryngeal nerve (RLN), a branch of the left vagus nerve, passes underneath the aortic arch in the thorax, hooking beneath the ligamentum arteriosum, the fibrous ligament between the aortic arch, and pulmonary artery. Compression of this nerve due to any thoracic pathology or damage during surgical interventions in the thorax may lead to vocal cord palsies manifested by hoarseness in patients. The common thoracic pathologies leading to left RLN palsy include (but are not limited to) the following: patent ductus arteriosus in adults and various cardiac diseases, including mitral stenosis, left ventricular failure, coronary artery disease, pulmonary arterial hypertension, aortic aneurysm, aortic lymph node enlargement in silicosis, and bronchogenic carcinoma involving the left upper lobe of the lung [1-7]. Previous researchers hypothesized that pulmonary arterial dilatation secondary to a wide range of cardiac disorders could be regarded as the single main factor directly responsible for left RLN palsy [1]. Again, the left RLN is considered to be at risk during esophageal resection surgeries, nodal dissection in lung cancer surgeries, and surgical closure of the patent ductus arteriosus in infants [1, 8-11]. Previous researchers tried to find an association between the RLN diameter and chances of injury during surgery [12]. Very limited literature is available providing the relation between the esophagus and the left RLN, as well as the normal diameters of the pulmonary artery and aorta in relation to the left RLN. If we can establish a data set regarding the safe distance of left recurrent laryngeal nerve from the esophagus, the usual range of diameters of this nerve and measurement of ligamentum arteriosum in healthy individuals that might be useful to prevent the inadvertent damage of this nerve during surgical procedures. Again, if a dataset having the usual diameters of pulmonary artery and aorta in healthy adults at close proximity of left RLN can be established, it could predict some disease processes at the beginning (with increased diameters) and predict the chances of compression of left RLN as well. A separate data set for the healthy male and female population might be more useful. The aim of this study is to measure the width of the left RLN in the thorax, the distance of the left RLN from the esophagus, and the diameter of the aorta and left pulmonary artery at the level of the ligamentum arteriosum and compare the values between males and females. In addition, we measured the length and width of the ligamentum arteriosum.

## Materials and methods

**Figure 1:**
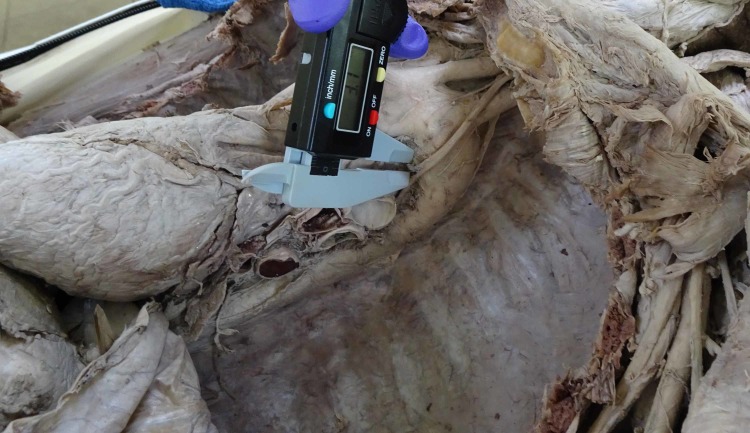
Showing the measurement of the left recurrent laryngeal nerve at its origin from the vagus nerve

This dissection-based, cross-sectional observational study was conducted at the Department of Anatomy, Medical University of the Americas, Saint Kitts and Nevis, over a period of two years. A total of 42 well-embalmed cadavers, which were available within the two year time period and were free from evident structural cardiovascular pathology, were examined for the study (20 males, 22 females). The ages of the cadavers ranged between 64 - 85 years, the majority were Caucasoid from the USA and no detailed medical history was available. The cadavers were used by medical students for gross anatomy dissection initially, so the thoracic cavities were already exposed using a standardized dissection technique. The cadavers with an intact recurrent laryngeal nerve, aorta, pulmonary trunk, ligamentum arteriosum, and esophagus were utilized for this research. The cadavers with considerable damage to the aforementioned structures were excluded from the study. A total of three cadavers were excluded because of previous damage to either the vagus nerve or pulmonary artery, so a total of 39 cadavers were finally included (19 males, 20 females) and measured in this study. The connective tissues covering the vagus and RLN were cleaned carefully to clearly expose the left RLN in the thorax, and the connective tissue around the aortic arch and pulmonary artery were also cleaned. The ligamentum arteriosum was exposed and secured with fine dissection. The esophagus was dissected underneath the aortic arch. The relation of the left vagus nerve with the three principal branches of the aortic arch (i.e., left subclavian, left common carotid, and brachiocephalic trunk) was noted. The width of the left RLN was measured at its origin from the vagus nerve at the lower border of the aortic arch (Figure 1).

**Figure 2:**
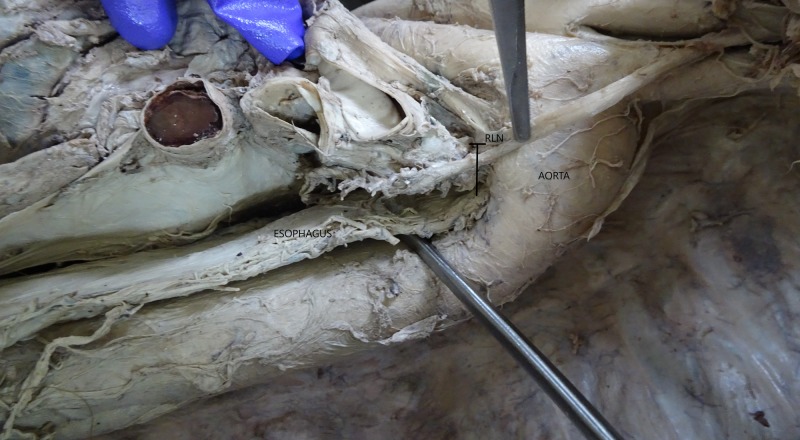
Showing the vertical relation between the left recurrent laryngeal nerve (RLN) and esophagus (structure with probe underneath)

The angulation between the left vagus nerve and left RLN was measured by a goniometer. The vertical distance between the left RLN and the esophagus was measured at the level of the aortic arch (Figure 2).

**Figure 3:**
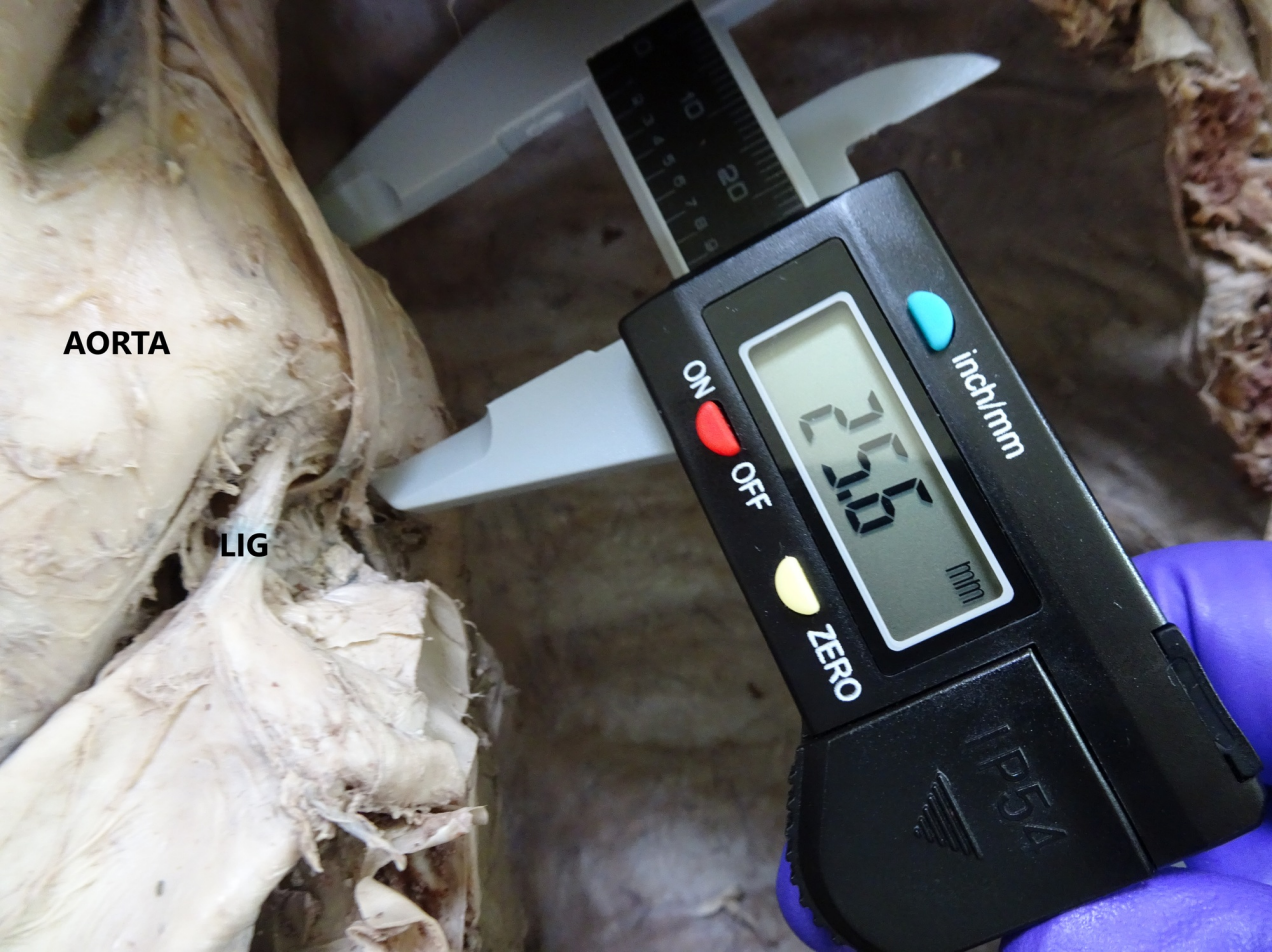
Showing the measurement of the diameter of the aortic arch distal to the ligamentum arteriosum (LIG) and vagus nerve

The diameter of the aortic arch distal to its attachment to the ligamentum arteriosum was recorded (Figure 3).

**Figure 4:**
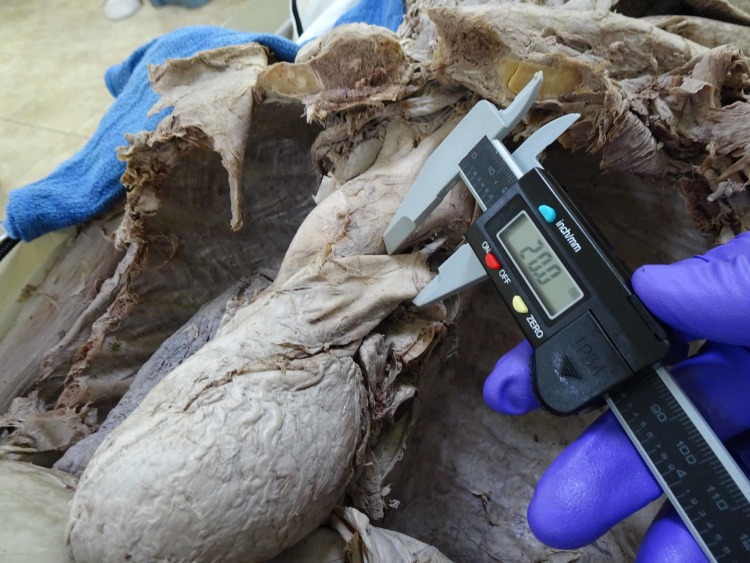
Showing the measurement of the pulmonary artery proximal to the attachment of the ligamentum arteriosum

The diameter of the pulmonary artery was measured proximal to its attachment to the ligamentum arteriosum (Figure 4).

**Figure 5:**
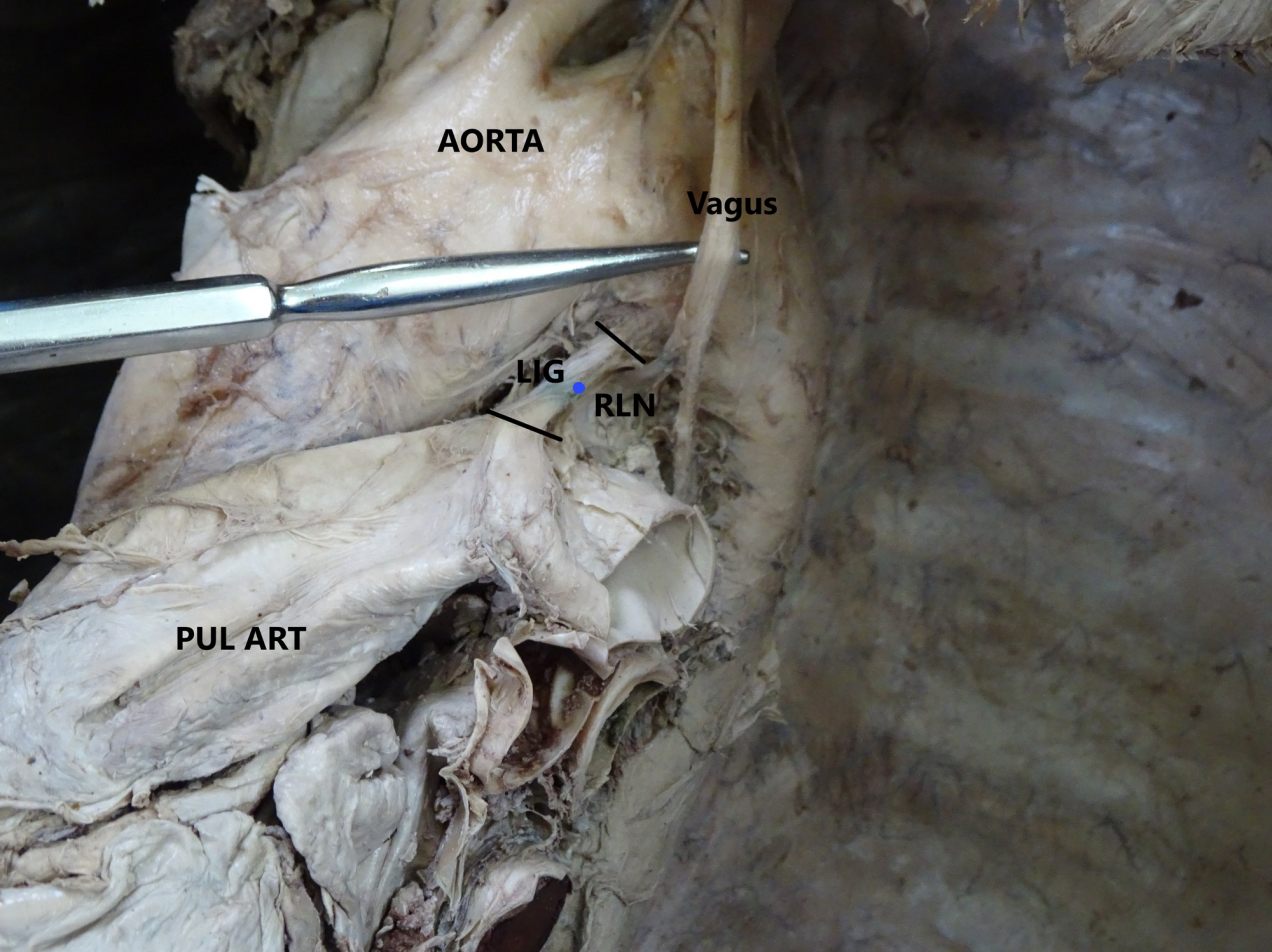
Showing the measurement of the ligamentum arteriosum LIG: ligamentum arteriosum; PUL ART: pulmonary artery; RLN: recurrent laryngeal nerve

The length of the ligamentum arteriosum was measured between its points of attachments from the undersurface of the aortic arch to the pulmonary artery, and the width of the ligamentum arteriosum was measured roughly at its midpoint (Figure 5).

All measurements were done by digital calipers. All the measurements were recorded and photographs were taken. The data analysis (calculation of mean and standard deviation) was done using Microsoft® Excel software (2013) (Microsoft® Corp., Redmond, WA) and p-values were elicited using t-test by an online p-value calculator [13].

## Results

In this study, 39 cadavers (19 males, 20 females) with an intact left vagus nerve, left RLN, and other structures under investigation were included and utilized for the measurements and data analysis. The left RLN made an angle of 5 to 7 degrees with the vagus nerve at its origin. The left RLN hooked underneath the aorta lying lateral to the ligamentum arteriosum in all cases and then coursed along the tracheoesophageal groove towards the larynx. It was seen lying 9.5 to 10.5 mm superficial to the esophagus in the superior mediastinum. The length of the ligamentum arteriosum varied across the cadavers. The data related to various parameters have been provided in Table 1.

**Table 1:** Showing the Average Values of Various Parameters in All Cadavers RLN: recurrent laryngeal nerve; SD: standard deviation

Parameters (mm)	Mean (n = 39)	SD
Width of the RLN	1.75	0.41
Distance of the RLN from the esophagus	9.88	0.31
Diameter of the aorta	26.14	2.43
Diameter of the pulmonary artery	19.93	1.08
Ligamentum arteriosum - length, width	15.89, 2.79	4.64, 0.64

No obvious difference was noted in the parameters among male and female cadavers (Tables 2-3). In addition, the left vagus nerve was seen to pass over the left subclavian artery in 80% of the cadavers. In 15%, the nerve passed along the medial border of the left subclavian artery, and in 5% of the cases, the vagus nerve crossed midway between the left common carotid and left subclavian artery while entering the thorax.

**Table 2:** Showing the Comparison Between Male and Female Values RLN: recurrent laryngeal nerve; SD: standard deviation

Parameters (mm)	Male (n=19)			Female (n = 20)			Observation
	Mean	SD	Range	Mean	SD	Range	
Width of the RLN	1.85	0.38	1.5 - 3	1.65	0.43	1.2 - 3	P = 0.08 Not clinically significant
Distance of RLN from the esophagus	9.87	0.3	9.5 - 10.5	9.9	0.33	9.5 - 10.5	P = 0.77 Not clinically significant
Diameter of the aorta	26.78	2.35	21.7 - 30	25.5	2.5	20.4 - 28.5	P = 0.1 Not clinically significant
Diameter of the pulmonary artery	19.71	1.15	17 - 21	20.15	1.0	19 - 21.6	P = 0.21 Not clinically significant

**Table 3:** Showing the Measurement Values of Ligamentum Arteriosum in Male and Female Cadavers SD: standard deviation

Ligamentum arteriosum (mm)	Male			Female			Observation
	Mean	SD	Range	Mean	SD	Range	
Length	16.83	4.62	7 - 27	14.95	4.65	6 - 25	P = 0.21 Not clinically significant
Width	2.98	0.75	2.2 - 4.5	2.59	0.52	2 - 3.5	P = 0.07 Not clinically significant

## Discussion

The current study provides a set of measurement values, including the left RLN diameter, the diameter of the aorta and pulmonary trunk at the level of attachment to the ligamentum arteriosum, the length and width of the ligamentum arteriosum, and the vertical distance of the left RLN trunk from the esophagus in the superior mediastinum.

In this study, we found the mean diameter of the left RLN was 1.75 mm (± 0.41) in cadavers, while in a study by Saito et al., the median size of this nerve was found to be 1.51 mm in living patients [12]. The difference in study methodology (cadaveric measurements in the present study and digital video recording, while surgical procedure in living esophageal cancer patients in the study by Saito et al.), way of measurement (digital caliper measurement versus calculating ratio between scissor size and nerve size), difference in ethnicity, and the size of the study population could be some possible causes of this reported difference. Vocal cord palsies are quite common in esophageal resection surgeries [8, 14]. According to Saito et al. [12], the width of the left RLN can be regarded as a predictor of post-surgical vocal cord palsies in esophageal resections. They found that a thin left RLN (having a diameter less than 1.5 mm) should be regarded as a high-risk factor in these surgeries. Amer suggested that a complete exposure of the left RLN during nodal dissection surgeries might the best possible way to avoid its damage [9]. The current study found the average vertical distance of the left RLN from the esophagus was 9.88 mm (± 0.31) when the nerve returns back towards the larynx along the tracheoesophageal groove. Edwards and McCullagh reported that in 95% of the cases the distance between the RLN and the esophagus was less than 1 cm, which corroborates with our findings [8].

**Figure 6:**
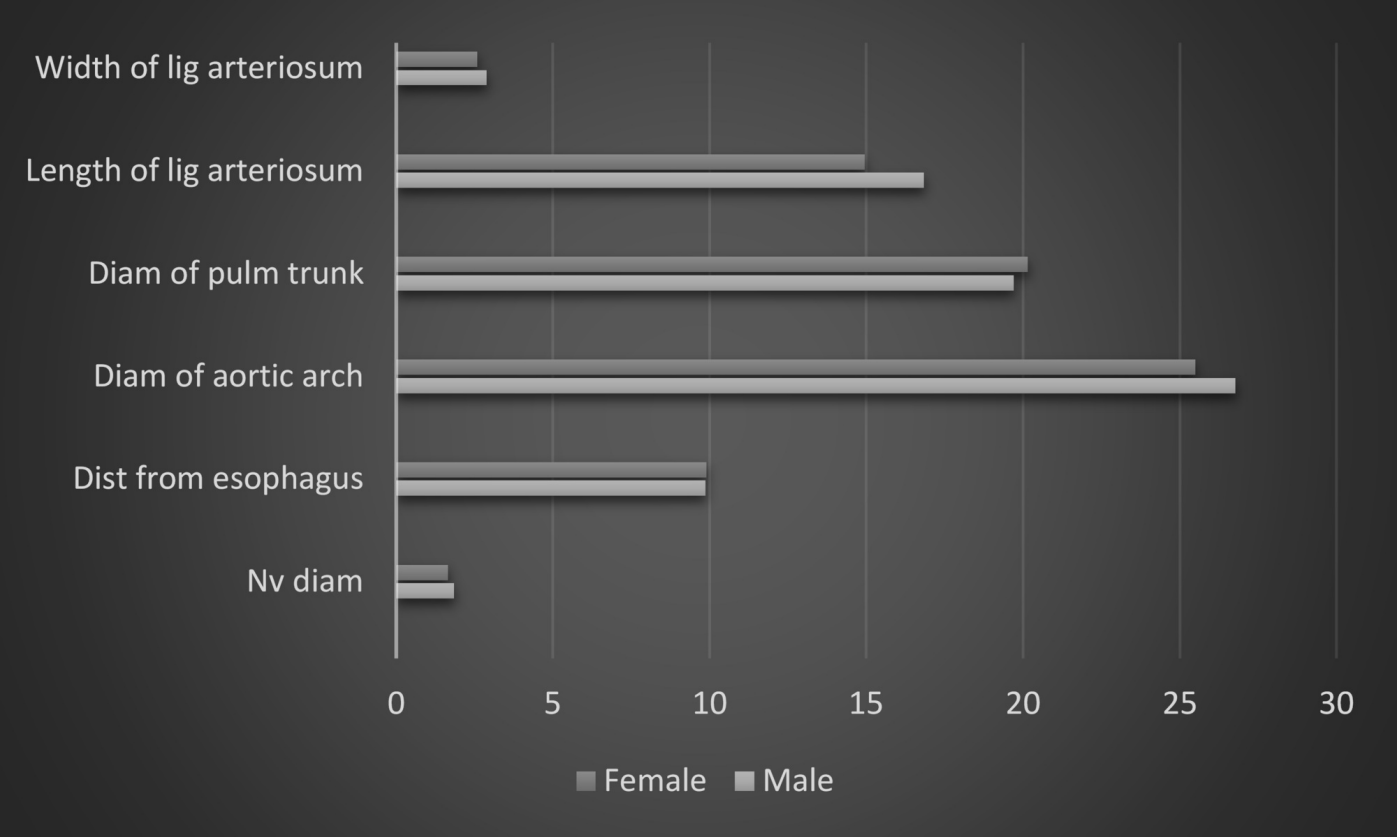
Showing a comparison between male and female values x-axis represents measurements in millimeters Diam: diameter; Dist: distance; lig: ligamentum; Nv: recurrent laryngeal nerve; Pulm: pulmonary

In the current study, we found the average diameter of the aortic arch was 26.14 ± 2.43 mm just distal to the attachment of the ligamentum arteriosum. An aortic aneurysm may lead to vocal cord palsy by compressing the left RLN; again, left RLN damage and vocal cord palsy may set in as a sequela of thoracic aortic aneurysm repair surgery [5]. In a study by Lee et al., the average diameter of the ascending aorta recorded by thoracic imaging was 30.7 mm and 32.8 mm in female and male subjects, respectively [15]. They concluded factors, including sex, systemic diseases, and lifestyle, do influence the aortic diameter. In our study, the diameters of the aortic arch in male and female cadavers were 26.78 and 25.5 mm, respectively. Although male cadavers showed relatively higher values in most of the parameters, no clinically significant difference was observed (Figure 6). Selecting different parts of the thoracic aorta for measurements could be responsible for the discrepancy in the findings among the current study and the study by Lee et al. In the present study, the average diameter of pulmonary artery just distal to the attachment of the ligamentum arteriosum was found to be 19.9 ± 1.08 mm. Lee et al. reported the average diameter of the main pulmonary artery was 26 ± 3.4 and 27 ± 3.4 mm, respectively, in female and male participants [15]. The difference of the findings could be contributed by (but not limited to) the following: different anatomical sites of measurements and post-embalming tissue shrinkage in cadavers [16]. Kuryama et al. suggested that computed tomography (CT) scan measurements of the pulmonary arterial diameter could be useful in detecting pulmonary arterial hypertension (HTN) and could be utilized to estimate the pulmonary arterial pressure [17]. Lange et al. suggested that pulmonary arterial diameter measured by thoracic CT could even predict borderline pulmonary hypertension and plan early diagnosis of underlying pathologies [18].

In the present study, the left RLN was found lateral to the ligamentum arteriosum in all cases. However, previous studies reported the location of ligamentum arteriosum might not be constant, and in rare cases, it could be located medial to the ligamentum arteriosum due to variation in embryonic development [19-20]. In our study, the average length and width of the ligamentum arteriosum were 15.89 ± 4.64 and 2.69 ± 0.64 mm, respectively. In a study by Keet et al., the length and width of the ligament were reported as 14.65 ± 7.33 mm and 2.86 ± 0.87 mm, respectively, which are very close to our findings. The length of the ligamentum varied from 6 to 27 mm across our study population, whereas Keet et al. reported the range as 3.48 - 31.48 mm [20].

The major limitation of this study is the small sample size and inability to know if any of these persons suffered from left RLN palsies and cardiac disorders. The second limitation is possible embalming-related tissue shrinkage and alteration of the venous size which might limit the clinical comparability of the recorded data. Future studies should be performed in a larger population and measurements should be compared between the normal population and the population with vocal cord palsy. A study on the pediatric population would be useful to provide guidelines for pediatric surgeries.

## Conclusions

In conclusion, we found the mean diameter of the left RLN was 1.75 mm, the mean distance of the nerve from the esophagus was 9.88 mm. The mean diameter of the aortic arch and the pulmonary artery just distal to the attachment of ligamentum arteriosum was 26.14 and 19.93 mm, respectively. The length and width of the ligamentum arteriosum were 15.89 and 2.79 mm, respectively. The range of measurements involving the recurrent laryngeal nerve, esophagus, aortic arch, pulmonary trunk, and ligamentum arteriosum might be useful for clinicians when investigating the underlying causes of left RLN palsy or while performing surgical procedures in close proximity to the left RLN in order to avoid damage to this nerve.
